# Hospitalisation by ambulatory care sensitive conditions at Rajavithi hospital, Bangkok

**DOI:** 10.1186/1471-2458-14-S1-P23

**Published:** 2014-01-29

**Authors:** Weena Promprasert, Nilawan Upakdee, Pudtan Phanthunane, Supasit Pannarunothai

**Affiliations:** 1 Department of Pharmacy, Rajavithi Hospital, Bangkok 10400, Thailand; 2 Faculty of Pharmaceutical Sciences, Naresuan University, Phitsanulok 65000, Thailand; 3 Faculty of Business, Economics and Communications, Naresuan University, Phitsanulok 65000, Thailand; 4 Faculty of Medicine, Naresuan University, Phitsanulok 65000, Thailand

## Background

This research aimed to study the trend of avoidable hospitalisation by ambulatory care sensitive conditions (ACSC) related to diabetes mellitus (DM) and hypertension (HT) in the universal coverage scheme (UC) patients at Rajavithi hospital.

## Materials and methods

A retrospective study collected data from electronic medical record from Rajavithi hospital and National Health Security Office. ACSCs selected were DM, HT and related diseases based on diagnosis codes (ICD-10) from fiscal year 2007–2011. The outcome measurements were number of patients, inpatient admissions, length of stay and cost from hospitalisation. The referral cases were excluded. Descriptive statistics were expressed as a median, 25th and 75th percentile, and percentage. The rates of ACSC were shown by trend line and R^2^ for a perfect linearity. The ACSC rate was calculated by number of admission patients on the condition divided by number of patients of that condition visited ambulatory care.

## Results

The average ACSC rate for DM was 19.5 per 1,000 DM patients and for HT was 3.5 per 1,000 HT patients. The rate for UC patients was higher than for other schemes (54.5 per 1,000 in DM, 10.3 per 1,000 in HT). The most common related procedures in DM were diabetic wound debridement and amputation of toe. Both DM and HT admissions occurred among the elderly of 60 to 74 years. The ratio of male to female was not different when compared to ambulatory patients in the same conditions (1.6 and 1.7 in DM and 0.7, 0.6 in HT). The hospitalisations at other regional hospitals were more than at Rajavithi hospital (19.3 vs. 3.9 per 1,000 in DM, 5.3 vs. 0.9 per 1,000 in HT). Median length of stay in DM and related diseases were 3 and 2 days, and in HT and related diseases were 2 and 6 days. The costs from hospitalisations by DM and HT increased as of the rise of admitted patients. The trend of the ACSC rate in UC patients increased only in DM related conditions (R^2^ = 0.84) whereas the rates decreased in DM, HT and HT related conditions (R^2^ = 0.29, 0.4, 0.02).

## Conclusions

The trend of ACSC rate in DM, HT and HT related conditions decreased while it increased in DM related disease conditions. ACSC rate in other regional hospitals were higher than Rajavithi hospital. Further study should investigate explanatory reasons.

